# A Child with Rabbits’ Eyes

**Published:** 1886-07

**Authors:** 


**A Child with Rabbit's Eyes.**—Two months ago Dr. Smith, of the Eye and Ear Department of the County Hospital, performed a difficult surgical operation on the eyes of a little three-month-old child, which was totally blind. It suffered from malignant opthalmia and the cornea of one eye was terribly bulged and completely opaque. Dr. Smith dissected out the inflamed cornea and ingrafted with delicate needles the cornea of a healthy rabbit. In performing the operation care was taken to preserve the delicate conjunctiva of the rabbit eye. No hope was expressed at the time that the transparency of the cornea would be preserved after it had been ingrafted. After a month had passed and the operation had proven successful in every other way there were some signs that the child might receive at least partial sight. So a similar operation was performed on the child's second eye, the corner of the rabbit's eye being first laid on a piece of cork and the membranes adjoining it, which had sloughed off after the first operation being removed, the surgeon being convinced from the first operation that the vitalizing power did not proceed from these membranes The eye on which the first operation was made has assumed its natural shape and color and the other is healing satisfactorily. They are normal in every respect, except the giving of sight. But for the operation the eyeballs would have burst.

